# Clinical identification of expressed proteins in adrenal medullary hyperplasia detected with hypertension

**DOI:** 10.3389/fendo.2022.1014366

**Published:** 2022-12-13

**Authors:** He Ma, Ke Wang, Bingjie Lai, Xueyan Zhang, Yang Lv, Ranwei Li

**Affiliations:** ^1^ Department of Anesthesiology, The Second Hospital of Jilin University, Changchun, China; ^2^ Department of Respiratory Medicine, The Second Hospital of Jilin University, Changchun, China; ^3^ Department of Intensive Care Unit, The Second Hospital of Jilin University, Changchun, China; ^4^ Faculty of Nursing, Jilin University, Changchun, China; ^5^ Department of Urinary Surgery, The Second Hospital of Jilin University, Changchun, China

**Keywords:** adrenal medullary hyperplasia, hypertension, proteomics, PNMT, MPZ, RAB3C, CD36

## Abstract

**Background:**

Hypertension remains a challenging public health problem worldwide, and adrenal gland-related diseases are one class of the major causes for secondary hypertension. Among them, one relatively rare pattern is adrenal hyperplastic hypertension caused by adrenal medullary hyperplasia (AMH), leading to excessive secretion of autonomic catecholamine. Given that the pathological changes of adrenal medulla are not well correlated to the onset and even severity of secondary hypertension, the molecular basis why some AMH patients are accompanied with hypertension remains unclear and is worth exploring.

**Aims:**

For this reason, this study aims at investigating differentially expressed proteins in clinical AMH tissue, with special focus on the potential contribution of these differentially expressed proteins to AMH development, in order to have a better understanding of mechanisms how AMH leads to secondary hypertension to some extent.

**Methods and results:**

To this end, AMH specimens were successfully obtained and verified through computed tomography (CT) and haematoxylin-eosin (HE) staining. Proteomic analyses of AMH and control tissues revealed 782 kinds of differentially expressed proteins. Compared with the control tissue, there were 357 types of upregulated proteins and 425 types of downregulated proteins detected in AMH tissue. Of interest, these differentially expressed proteins were significantly enriched in 60 gene ontology terms (P < 0.05), including 28 biological process terms, 14 molecular function terms, and 18 cellular component terms. Pathway analysis further indicated that 306 proteins exert their functions in at least one Kyoto Encyclopedia of Genes and Genomes (KEGG) pathway. Western blotting showed enhanced expression of phenylethanolamine N- methyltransferase (PNMT), myelin protein zero (MPZ), and Ras-related protein Rab-3C (RAB3C), and reduced expression of cluster of differentiation 36 (CD36) observed in AMH tissue in comparison with controls.

**Conclusions:**

Clinical AMH specimens display a different proteomic profile compared to control tissue. Of note, PNMT, MPZ, RAB3C, and CD36 are found to differentially expressed and can be potential targets for AMH, providing a theoretical basis for mechanistic exploration of AMH along with hypertension.

## Introduction

Hypertension remains an unsolved public health problem worldwide, with an estimated prevalence of 26%, and about 5%-10% for secondary hypertension (1, 2). Adrenal gland-related disorders, as exemplified by adrenal adenoma and primary aldosteronism, are well recognized as major causes of secondary hypertension (3, 4). In addition, adrenal medullary hyperplasia (AMH), as a benign but rare pathological change, can result in secondary hypertension as well (5, 6). Even though the association between AMH and hypertension is not clearly investigated based on current literatures (7, 8), it is still necessary to study this rare histological change of adrenal gland detected with hypertension simultaneously. Nevertheless, there has been a lack of relevant research reports about this topic. Therefore, basic studies of AMH using multiple approaches may extend our current understanding of secondary hypertension in terms of mechanisms.

On the one hand, measuring specific protein markers can provide functional insights into molecular mechanisms of disease progression and corresponding biological processes, and further these markers may have potential value for disease diagnosis and treatment (9). On the other hand, the major goal of proteomics research is to identify novel proteins that can be used as disease markers and/or therapeutic targets (10–12). Current proteomics approaches can reliably identify and quantify differentially expressed proteins (13, 14). At the beginning, most of researchers applied quantitative proteomics to determine the relative levels of proteins in diseased tissues in comparison with controls. Nowadays, the absolute quantities are additionally measured to characterize more comprehensive biological states, providing a deeper understanding of proteome dynamics as well as the identification and validation of novel protein biomarkers (15). Given its advantages, for example high quantitative accuracy, high throughput, and no limitation of sample sources, isobaric tags for relative and absolute quantitation (iTRAQ), as a powerful technique, has been extensively applied to measure the relative and absolute expression of differentially expressed proteins using internal peptide standards in different disease settings, such as infection, aging and tumours (16–19).

As introduced in the above part, it is intriguing to profile differentially expressed proteins in AMH tissue using iTRAQ. So far, there have been no available proteomic studies about AMH with secondary hypertension yet. For this reason, the present study mainly utilises quantitative iTRAQ analysis to compare the proteome profile of clinical AMH tissues with normal adrenal medulla tissue, screen out differentially expressed proteins, and anticipate their possible functions and involved pathways in the context of AMH, in order to acquire more knowledge about the association between AMH and hypertension.

## Materials and methods

### Patient recruitment

Twenty-four patients who were diagnosed with adrenal medullary hyperplasia (AMH) *via* computed tomography (CT) and underwent adrenal resection in the Second Hospital of Jilin University from June 2010 to April 2019 were included. Each patient has signed the written informed consent form before participation, and this study was approved by the Ethics Committee of our hospital. Clinical specimens were directly collected from these patients during surgery, and pathologically confirmed as AMH *via* HE staining performed by senior pathologists. Additionally, all enrolled patients were clinically diagnosed with hypertension. Importantly, all patients did not receive any adjuvant therapy before surgery. Specific inclusion criteria were summarized as follows (20): preoperative presentation of hypertension; excess catecholamine production; CT scan results showing hyperplastic changes in the adrenal medulla, and that hyperplastic adrenal medulla has extended to the alae and tail of adrenal gland; excised adrenal tissue pathologically diagnosed as AMH; and return of blood pressure to the normal level after surgery.

### Tissue collection

The excised adrenal gland was directly sent to Pathology Department after removal, which was ready for detailed analyses by senior pathologists. Adrenal cortex and medulla are distinct by eye due to their different colours and positions. A small part of fresh tissue was used for pathological diagnosis. After that, normal adrenal cortex, normal adrenal medulla and AMH diseased samples were carefully isolated from the remaining tissue, which were frozen at −80°C for further analysis. All specimens including AMH diseased samples and control adrenal medulla samples in this study were isolated from the same cohort of patients, which were determined by pathological diagnosis. Twenty of these specimens were used for proteomic analysis, and the rest four were used for subsequent validation by Western blotting.

### iTRAQ quantitative analysis

100 mg of specimens were obtained from both diseased and control samples of twenty AMH patients, and 20 pieces of AMH specimens and 20 pieces of control specimens were mixed separately. Homogenates were collected and total proteins were extracted by SDT lysis buffer. The standard procedures were described as follows: the sample was lysed by 1× SDT buffer, and then transferred to 2 mL tubes with quartz sand. The lysate was homogenized twice at the speed of 6.0 m/s for 30 sec, and the homogenate was boiled for 15 min. After centrifugation at 14,000 g for 40 min, the supernatant was filtered through a 0.22 µm filter. The protein concentrations were determined using BCA Protein Assay Kit (Beyotime Biotechnology, Shanghai, China), and samples were stored at −80°C.

Proteins were enzymatically decomposed into peptides using trypsin buffer, and 100 μg of peptides were extracted from each specimen and labeled according to instructions of the AB SCIEX’s iTRAQ^®^ Simplex-4plex Applications Kit (iTRAQ^®^). In brief, the sample was centrifuged at 12,500 g for 25 min. 30 μL was removed, and dithiothreitol (DTT) was added until the final concentration was 100 mM. The sample was boiled for 5 min, and 200 μL of UA buffer and 100 μL IAA buffer were added. The sample was oscillated at 600 rpm for 1 min, incubated at room temperature (RT) for 30 min, and was then centrifuged followed by adding 100 μL of UA buffer. Next, 100 μL of dissolution buffer, and 40 μL of trypsin buffer were added, and samples were then maintained at 37°C for 16 h to 18 h. After centrifugation, 20 μL of dissolution buffer was added, the filtrate was collected, and the concentration was measured using Nano Drop 2000 (Thermo Fisher Scientific).

Next, protein labeling was performed. ITRAQ reagent was added to 100 μg of enzymatic hydrolysates and incubated at RT for 2 h. Then, 100 μL water was added to terminate the reaction. All labeled samples were mixed, swirled, and centrifuged. After grading using the Agilent 1260 Infinity II HPLC system, the labeled peptides were mixed and separated using the Easy nLC system. Later on, samples were separated by chromatography and analyzed using Q Exactive Mass Spectrometer. Proteome Discoverer 2.1 were used for database identification and quantitative analysis.

### Bioinformatics analyses

Gene Ontology (GO) analysis was conducted to identify the biological functions of differentially expressed proteins, including biological process (BP), molecular function (MF), and cellular component (CC) (http://www.geneontology.org) (21). In addition, Kyoto Encyclopedia of Genes and Genomes (KEGG) pathway mapping (http://www.genome.jp/kegg) was applied to analyse enriched pathways of different protein clusters (22). STRING version 10.1 (http://string-db.org) was used to identify protein-protein interaction (PPI) networks (23), which were defined by the presence of either direct/physical or indirect/functional associations.

### Western blotting

To verify the differential expression of these four proteins in AMH, Western blotting was performed using four pairs of specimens from the same patients. These proteins were extracted and separated using 15% SDS-PAGE precast gel, and then transferred onto PVDF membranes (Millipore, Bedford, MA, USA). These membranes were blocked in 5% BSA-Tris-buffered saline with 0.1% Tween (TBST) at RT for 1 h, and then incubated with different primary antibodies at 4°C overnight, including anti-MPZ (10572-1-AP, Proteintech), anti-RAB3C (10788-1-AP, Proteintech), anti-PNMT (13217-1-AP, Proteintech), anti-CD36 (10572-1-AP, Proteintech) and anti-β-Actin (66009-1-Ig, Proteintech). On the second day, the membranes were washed for three times using TBST and incubated at RT for 1 h with corresponding secondary antibodies linked to horse radish peroxidase (HRP). Followed by three times of washing, proteins bands were visualized by LICOR machine and protein densities were quantified using ImageJ software and normalised to β-Actin.

### Statistical analysis

Data are expressed as means ± standard deviations. GraphPad Prism 9 Software (San Diego, CA, USA) was used for statistical analysis. A *P*-value below 0.05 was considered as statistically significant.

## Results

### Clinical diagnosis and pathological confirmation of AMH

**Figure 1:**
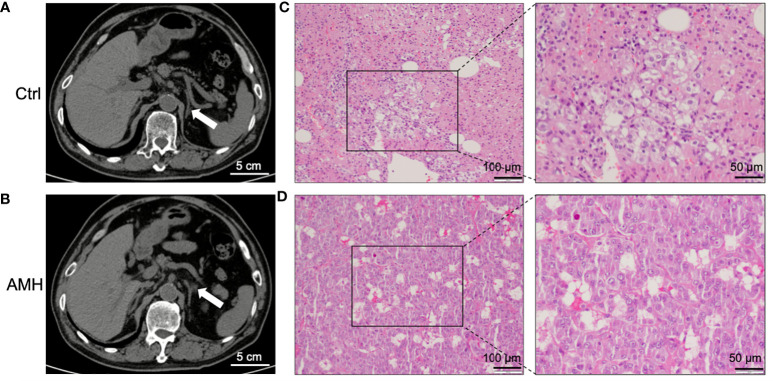
Clinical diagnosis and pathological verification of AMH. **(A, B)** Representative computed tomography (CT) scan images of control adrenal tissue **(A)** and AMH tissue **(B)**. **(C, D)** Representative haematoxylin-eosin (HE) staining images of control adrenal tissue **(C)** and AMH tissue **(D)**.

General information of twenty-four patients included in this study was listed in **Table 1**. The main clinical feature of these AMH patients was hypertension, as demonstrated in **Table 1**. CT scan images showed hyperplastic changes in the adrenal medulla (**Figure 1B**) in comparison with healthy controls (**Figure 1A**). Of interest, hyperplastic adrenal medulla has extended to the alae and tail of adrenal gland (**Figure 1B**), which is the main diagnostic criteria of AMH. In addition, representative HE staining images demonstrated chromatocyte morphology in both AMH and control (**Figures 1C, D**). Collectively, these obtained samples meet experimental requirements, and can be used for further analysis.

**Table 1:** Main characteristics of patients included in this study.

Patient number	Gender	Age	Weight (kg)	BP (mmHg)	Postoperative pathological diagnosis
1	female	62	67	160/100	AMH (left)
2	male	68	79	164/92	AMH (left)
3	female	64	70	220/120	AMH (right)
4	female	33	70	160/110	AMH (right)
5	male	54	70	170/110	AMH (left)
6	male	59	70	180/105	AMH (right)
7	female	41	64	200/110	AMH (right)
8	male	35	80	180/110	AMH (right)
9	male	63	40	197/103	AMH (left)
10	female	46	85	220/130	AMH (right)
11	male	64	70	177/104	AMH (right)
12	female	38	60	180/110	AMH (left)
13	female	48	60	200/110	AMH (right)
14	male	55	71	190/120	AMH (right)
15	female	63	70	200/101	AMH (left)
16	female	59	68	186/112	AMH (left)
17	female	54	75	180/110	AMH (right)
18	female	28	55	170/115	AMH (right)
19	male	56	87	170/110	AMH (left)
20	male	46	94	230/130	AMH (left)
21	male	77	70	160/83	AMH (right)
22	male	64	72	170/90	AMH (left)
23	male	38	94	170/130	AMH (left)
24	female	56	65	175/105	AMH (left)

### Identification of differentially expressed proteins in AMH

After clear diagnosis of AMH, iTRAQ was then applied to compare the abundance of proteins in AMH tissues and adjacent control adrenal tissues from 20 patients included in this study. These proteins could be considered as differentially expressed if their expression differences were more than 20% or less than 20% between two groups, with *P* < 0.05. Our results indicated that there were 782 types of differentially expressed proteins, with 357 upregulated proteins and 425 downregulated proteins in AMH tissues. The top 35 candidates of the most upregulated proteins included Chromogranin-A (CHGA), phenylethanolamine N-methyltransferase (PNMT), myelin protein zero (MPZ, P0), and Ras-related protein Rab-3C (RAB3C). Among them, CHGA was the most upregulated protein, and its level was 4.22-fold higher in the AMH group compared with the control. The top 35 candidates of the most downregulated proteins included COX assembly mitochondrial protein 2 (CMC2), cluster of differentiation 36 (CD36). CMC2 was the most downregulated protein, and its level was 9.1-fold lower in the AMH group compared with the control.

### Gene ontology analysis

**Figure 2:**
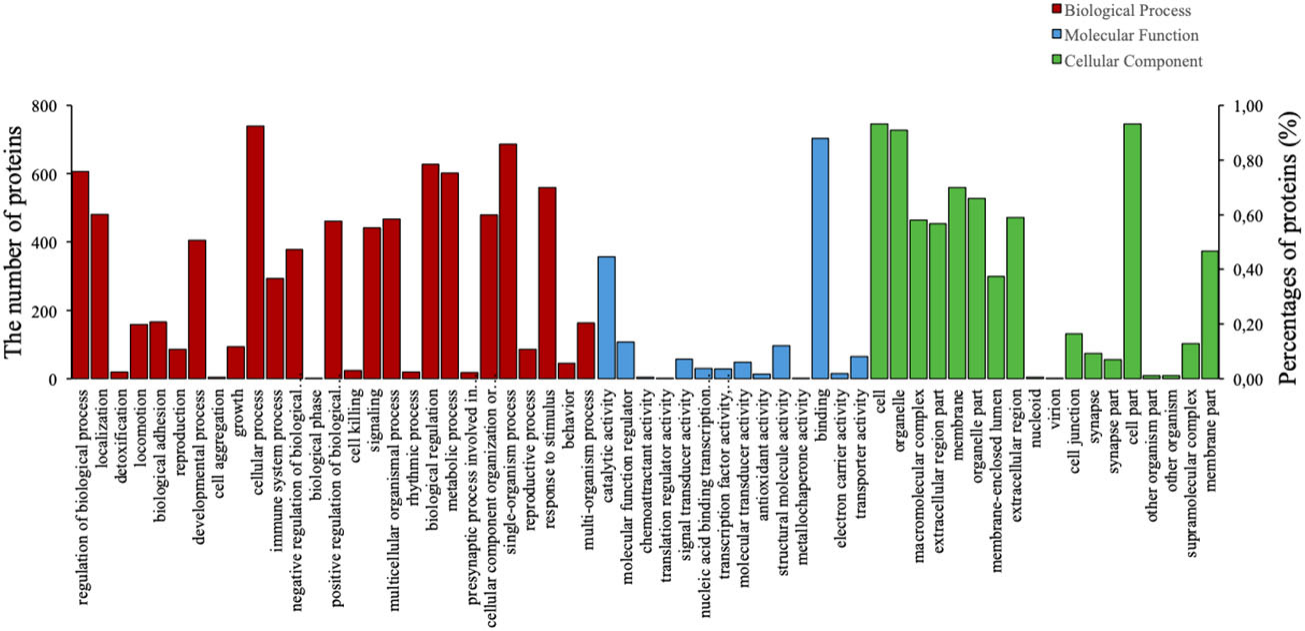
Gene Ontology (GO) analysis results of differentially expressed proteins in AMH tissues compared with adjacent control tissues. Analyses were performed using three terms: biological process (BP), molecular function (MF), and cellular component (CC).

**Figure 3:**
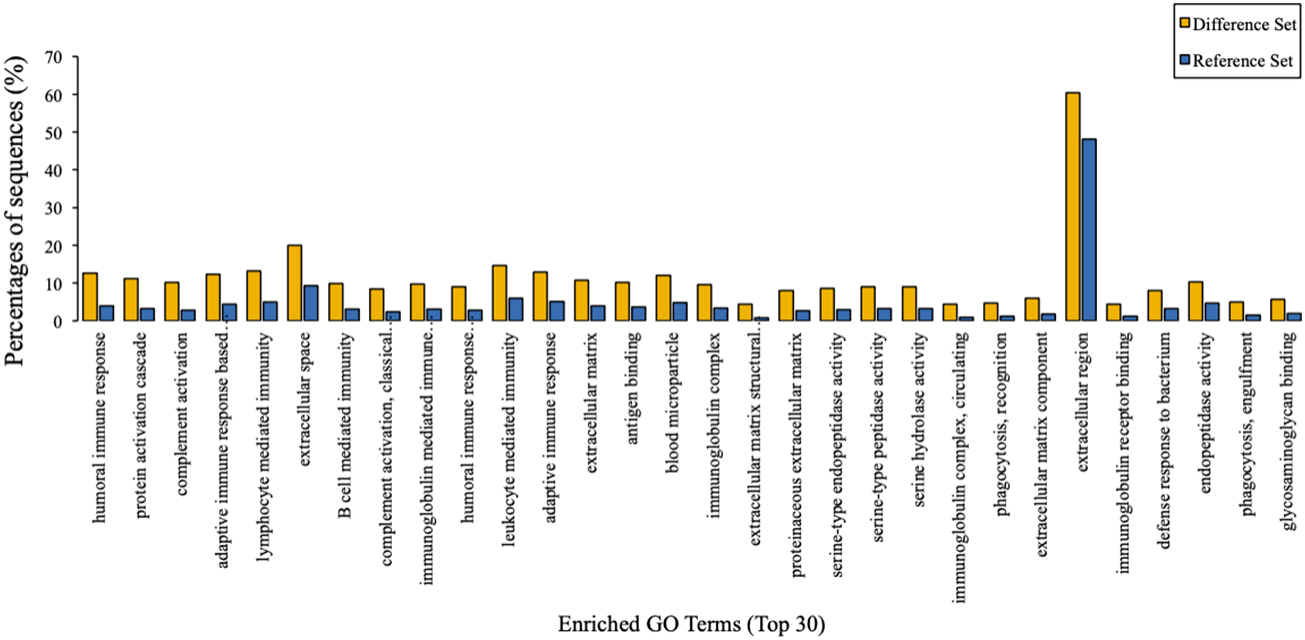
GO enrichment analysis results. The top 30 enriched GO terms of target proteins were displayed in comparison with background proteins. Difference set: target proteins; Reference set: background proteins.

To predict their probably involved functions, we then performed Gene Ontology (GO) analysis to characterize these differentially expressed proteins into three categories: biological process (BP), cellular component (CC), and molecular function (MF). **Figure 2** indicated that these differentially expressed proteins were significantly enriched in 60 GO terms (*P* < 0.05), encompassing 28 BP terms, 14 MF terms, and 18 CC terms. Seven enriched BP terms were ‘regulation of biological process’, ‘localisation’, ‘detoxification’, ‘locomotion’, ‘biological adhesion’, ‘reproduction’ and ‘developmental process’. Seven enriched MF terms were ‘catalytic activity’, ‘molecular function regulator’, ‘chemoattractant activity’, ‘translation regulator activity’, ‘signal transducer activity’, ‘nucleic acid binding transcription factor activity’ and ‘transcription factor activity, protein binding’. Seven enriched CC terms were ‘cell’, ‘organelle’, ‘macromolecular complex’, ‘extracellular region part’, ‘membrane’, ‘organelle part’ and ‘membrane-enclosed lumen’, as demonstrated in **Figure 2**. Moreover, we also identified 30 enriched GO terms, which showed the most significantly changes between AMH and controls, regarding three main functional modules (**Figure 3**).

### Kyoto Encyclopedia of Genes and Genome pathway analysis

**Figure 4:**
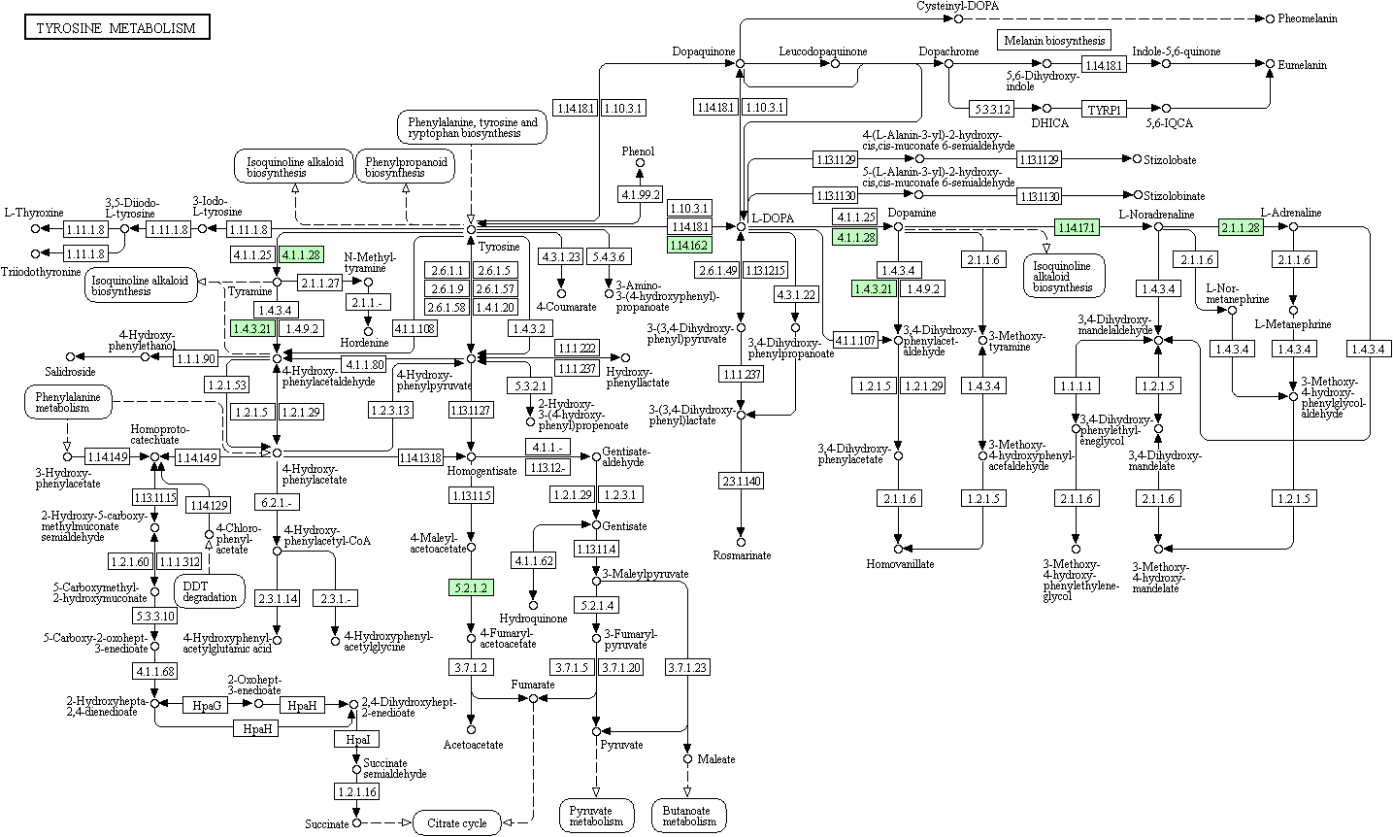
KEGG pathway functional analysis results. Major pathways include tyrosine 3-monooxygenase (EC:1.14.16.2), dopamine beta-monooxygenase (EC:1.14.17.1), phenylethanolamine N-methyltransferase (EC:2.1.1.28), tyrosine 3-monooxygenase (EC:1.14.16.2), aromatic-L-amino-acid/L-tryptophan decarboxylase (EC:4.1.1.28/4.1.1.105), maleylacetoacetate isomerase (EC:5.2.1.2), maleylacetoacetate isomerase (EC:5.2.1.2), and primary-amine oxidase (EC:1.4.3.21).

**Figure 5:**
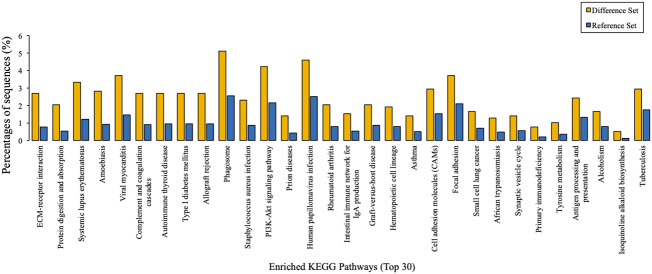
KEGG pathway enrichment analysis results. The top 30 enriched pathways of target proteins were displayed in comparison with background proteins. Difference set: target proteins; Reference set: background proteins.

Taking tyrosine metabolism as an example, KEGG metabolic pathway analysis suggested that 306 kinds of proteins exert their functions through at least this pathway (**Figure 4**). Furthermore, KEGG pathway enrichment analysis provided an overview of various pathways in which these differentially expressed proteins might be involved. In a similar vein, we also identified 30 enriched KEGG pathways, which showed the most significantly changes between AMH and controls, as exemplified by ‘ECM-receptor interaction’, ‘protein digestion and absorption’, and ‘systemic lupus erythematosus’ (**Figure 5**).

### Protein-protein interaction network of differentially expressed proteins

**Figure 6:**
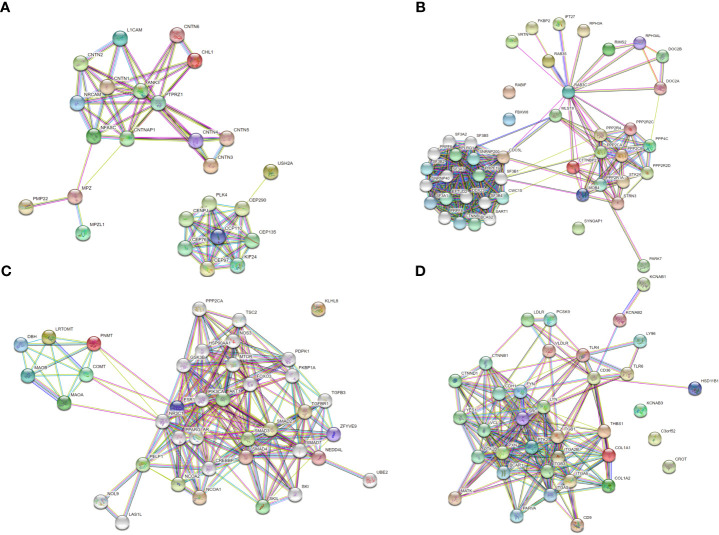
Protein-protein interaction (PPI) network based on differentially expressed proteins. Each node indicates an individual protein, red nodes indicate upregulated proteins in AMH tissue, and green nodes indicate downregulated proteins in AMH tissue. **(A)** PPI network based on upregulated MPZ.P0. **(B)** PPI network based on upregulated RAB3C. **(C)** PPI network based on upregulated PNMT. **(D)** PPI network based on downregulated CD36.

To further investigate specific regulatory mechanisms of AMH, protein-protein interaction (PPI) networks of these differentially expressed proteins were determined by using the Search Tool for the Retrieval of Interacting Genes/Proteins (STRING) database. **Figure 6** showed that four types of differentially expressed proteins, including PNMT, MPZ (P0), RAB3C, and CD36, displayed complex interactions with many other proteins, suggesting they may function in the pathogenesis of AMH through different mechanisms.

### Validation of differentially expressed proteins by Western Blotting

**Figure 7:**
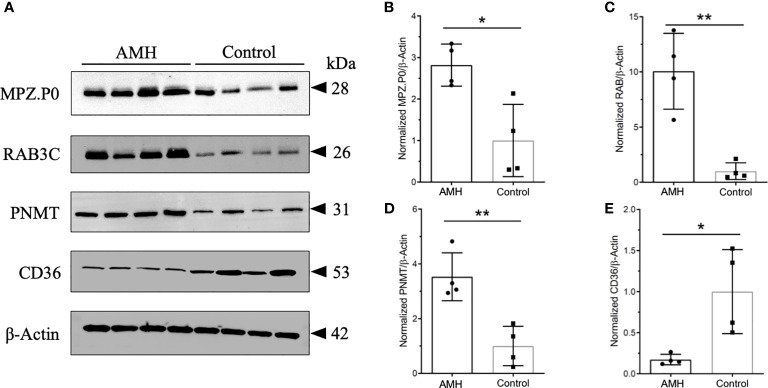
Representative Western blots and quantification results of four kinds of differentially expressed proteins. **(A)** These four proteins displayed distinctly different expression in AMH and control tissues. MPZ.P0, RAB3C, and PNMT were increased in AMH, whereas CD36 was decreased in AMH in comparison with controls. n = 4 for each group. β-Actin was used as the loading control. **(B)** Quantification results of MPZ.P0 normalized to β-Actin. **(C)** Quantification results of RAB3C normalized to β-Actin. **(D)** Quantification results of PNMT normalized to β-Actin. **(E)** Quantification results of CD36 normalized to β-Actin. * *P* < 0.05, ** *P* < 0.01.

We then performed Western blotting using AMH and control tissues to determine the relative levels of these four proteins identified in PPI networks. MPZ (P0), RAB3C, and PNMT were found to be increased, whereas CD36 was found to be decreased in AMH tissues in comparison with controls (**Figure 7**). There were a 2.82-fold increase of PNMT, a 10.06-fold increase of MPZ, a 6.27-fold increase of RAB3C, and a 5.81-fold decrease of CD36 in AMH samples compared to controls (**Figures 7B–D**). These data were consistent with the above-mentioned iTRAQ results, together suggesting their potential significance in AMH.

## Discussion

Given that there is a limited understanding of AMH-associated secondary hypertension (24, 25), this study mainly investigated the proteome profiles of AMH as well as adjacent control tissues using iTRAQ quantitative analysis, an advanced method with maximum protein coverage and precise quantitation (26–28). It turned out that 782 kinds of differentially expressed proteins were identified in AMH tissue, including 357 types of upregulated proteins and 425 types of downregulated proteins. Further bioinformatics analysis demonstrated that these differentially expressed proteins may have various molecular functions. Of special note, GO analysis results suggested significant enrichment of these differentially expressed proteins in 60 GO terms (*P* < 0.05), including 28 BP terms, 14 MF terms and 18 CC terms. Based on literatures, we chose several pivotal candidates to validate their changed expression on the protein level. In a similar vein, Western blotting results confirmed that PNMT, MPZ, and RAB3C were upregulated whereas CD36 was downregulated in human AMH tissues compared to controls. Additionally, several other potentially interesting differentially expressed proteins were also revealed by iTRAQ analysis. Even though we did not validate all these proteins, current results from this study would provide many important indications for future research. Here, we mainly focused on these four kinds of differentially expressed proteins, i.e. PNMT, MPZ, RAB3C, and CD36.

Although there are no relevant publications to report the involvement of these four differentially expressed proteins in AMH pathogenesis, some previous studies have demonstrated that these differentially expressed proteins are extensively implicated in other disease settings. The first candidate, MPZ, belongs to type I transmembrane proteins with an extracellular immunoglobulin-like domain (29). In the functional sense, MPZ affects cell adhesion and plays a role in homotypic interactions with other myelin protein zero proteins, which might contribute to neuropathy further (30, 31). Raasakka et al. reported that MPZ participates in the peripheral neuropathies of Charcot-Marie-Tooth (CMT) disease, even though the underlying mechanism how MPZ contributes to this disease has not yet been comprehensively studied (29). Next, RAB3C is a secreted RAB that participates in the regulation of secretory vesicle exocytosis and is generally expressed in peripheral blood mononuclear cells (PBMC) and platelets, as well as the nervous system, colon, ovaries and seminal vesicles. Chang et al. reported that upregulation of RAB3C promoted metastasis of colorectal cancer *via* enhancing IL-6 secretion as well as protein recruitment through STAT3-related pathways (32).

Then, PNMT is another differentially expressed protein that we verified in this study. PNMT is an adrenal enzyme that biosynthesizes epinephrine from norepinephrine, and a key determinant of epinephrine production in adrenal chromaffin cells upon acute and chronic stress (33, 34). Thus, PNMT is a marker of adrenergic cells (35). Nguyen et al. reported increased mRNA and protein levels of PNMT, as well as enhanced enzymatic activity in hypertensive rats, which led to increased adrenal activity and hypertension (34). Based on this, elevated PNMT levels may at least partially explain the occurrence of hypertension in AMH patients observed in our study, whose blood pressure return to the normal level after unilateral adrenal gland resection. At last, CD36 is a multifunctional receptor that is expressed in various cell types and tissues. CD36 binds to many ligands, including long chain fatty acids, thrombospondin, collagen, lipopolysaccharides and oxidized lipoproteins. Therefore, CD36 participates in a wide range of cellular processes, such as apoptosis, angiogenesis, and phagocytosis in addition to fatty acid uptake, and involves in diverse diseases, such as diabetes, atherosclerosis and hypertension (36, 37). Notably, Yang et al. found that blockade or knockout of CD36 in animal models prevented kidney injury (38). These above-mentioned studies together suggest that these four differentially expressed proteins exert potent functions in the initiation and progression of several different diseases. In this study, our proteomic analysis was the first original study to clearly demonstrate that PNMT, MPZ, RAB3C and CD36 levels were significantly changed in AMH tissues compared with control tissues. Nevertheless, these results provide a preliminary foundation for further research on AMH.

In addition to proteomic research of AMH described in the above part, some studies also reported transcriptional alterations in the context of adrenal diseases. An increasing body of evidence has described the genetics of primary aldosteronism, Cushing’s syndrome (CS), macronodular adrenocortical hyperplasia (MAH), and adrenocortical tumours (39–41). Vaduva and coworkers emphasized that several potent molecular mutations might contribute to the progression of CS and adrenal tumors, and they especially focused on *PRKAR1A* germline-inactivating alterations (41). Interestingly, they summarized other somatic mutations in aldosterone-producing adenomas, such as *ATP1A1*, *CACNA1D*, *ATP2B3*, *CTNNB1*, and *KCNJ5* (41). Almeida and colleagues demonstrated that MAH progression might involve chromosomes 20q13 and 14q23 based on chromosomal enrichment analysis (39). Moreover, Espiard and Bertherat systemically reviewed the gene alterations implicated in adrenal tumors and highlighted that recognition of these mutations would be beneficial to the development of more precise and efficient diagnostic and therapeutic tools (40). Collectively, emerging transcriptome and proteome studies will provide more important evidence for the underlying molecular mechanisms of AMH and many other endocrine disorders.

More recently, Mejia et al. gave a comprehensive review about AMH and hypertension, and especially addressed that AMH is rarely considered among the different causes of endocrine hypertension (42). This review confirms the clinical significance of our study in this field from the other perspective. Additionally, Ruiz-Manzanera et al. identified dopamine as a potential linker among AMH, Conn syndrome, and refractory hypertension (43). Furthermore, various coexistent disorders of AMH, such as cerebral angiomas and pheochromocytoma, make clinical consideration of AMH-associated hypertension more complicated (44–46). Taken together, our study would provide some promising candidates for AMH and kick off basic research of this topic.

## Conclusions

In summary, this study for the first time identifies specific differentially expressed proteins in human AMH tissues detected with secondary hypertension using proteomics approach, along with the verification of four proteins *via* Western blotting. Differentially expressed PNMT, MPZ, RAB3C, and CD36 can be potential targets for AMH, providing a theoretical basis for mechanistic exploration of AMH along with hypertension. In the meanwhile, several potential limitations of this study should be acknowledged. This study did not consider differences in protein expression among individual patients. Because iTRAQ labeling was performed by using pooled samples from two experimental groups. In addition, due to specimen limitations, only four kinds of proteins were validated in this study. Importantly, functional analyses of these differentially expressed proteins utilising either cell lines or animal models are essential to elucidate their roles in AMH. Even so, this study using iTRAQ-based laboratory method provides an important and initial understanding of AMH-associated secondary hypertension.

## Data availability statement

The datasets presented in this study can be found in online repositories. The names of the repository/repositories and accession number(s) can be found in the article/supplementary material.

## Ethics statement

The studies involving human participants were reviewed and approved by The Ethics Committee of the Second Hospital of Jilin University. The patients/participants provided their written informed consent to participate in this study.

## Author contributions

HM, RL, and KW conducted the literature search and wrote the paper. RL and KW designed the study, obtained funding, and provided technical support. HM, BL, XZ, and YL participated in the main experiments and collected the data. All authors read and approved the final manuscript.
